# Characterization of Cetacean Morbillivirus in Humpback Whales, Brazil

**DOI:** 10.3201/eid3006.231769

**Published:** 2024-06

**Authors:** Derek B. de Amorim, Laura J. de Camargo, Paula R. Ribeiro, Renata da F. Budaszewski, Jean Carlo O. Menegatt, Milena C. Paz, Lucas T. de Castro, Paula R. Almeida, Juliana C. Olegário, Cláudio W. Canal, Luciana Sonne

**Affiliations:** Universidade Federal do Rio Grande do Sul, Imbé, Brazil (D.B. de Amorim);; Universidade Federal do Rio Grande do Sul, Porto Alegre, Brazil (L.J. Camargo, P.R. Ribeiro, R.F. Budaszewski, J.C.O. Menegatt, M.C. Paz, L.T. de Castro, J.C. Olegário, C.W. Canal, L. Sonne);; Universidade FEEVALE–Campus II, Novo Hamburgo, Brazil (P.R. Almeida)

**Keywords:** Cetacean morbillivirus, humpback whales, Guiana dolphin morbillivirus, marine mammal, epizootic, CeMV, mysticete, viruses, Brazil

## Abstract

Cetacean morbillivirus is an etiologic agent associated with strandings of live and dead cetacean species occurring sporadically or as epizootics worldwide. We report 2 cases of cetacean morbillivirus in humpback whales (*Megaptera novaeangliae*) in Brazil and describe the anatomopathological, immunohistochemical, and molecular characterization findings in the specimens.

The humpback whale (*Megaptera novaeangliae*) is a mysticete with a cosmopolitan distribution, including Brazil (*1*). Morbillivirus in cetaceans first occurred in 1988 in Europe. Since then, various strains of cetacean morbillivirus (CeMV) have been associated with strandings of live and dead cetaceans worldwide (*2*). 

Two humpback whale specimens (MN1, MN2) stranded alive in southern Brazil in 2022 (Table; Appendix Figure 1). Because of deteriorated health, the whales were euthanized. In both cases, anesthetic protocols were performed by intramuscular infusion, followed by intracardiac administration of potassium chloride. 

**Table:** Stranding data from 2 humpback whale specimens in southern Brazil, 2022*

Stranding data	MN1	MN2
Stranding date	September 14, 2022	October 5, 2022
Stranding location	Mostardas, RS	Jaguaruna, SC
Geographic coordinates	30°49′34.4″S, 50°33′59.2″W	28°45′48.6″S, 49°07′38.9″W
Carcass condition	Fresh	Fresh

*MN, *Megaptera novaeangliae*; RS, Rio Grande do Sul State; SC, Santa Catarina state.

We performed necropsies immediately after euthanasia, fixed organ samples in 10% formalin for histologic analysis, and froze samples at −20°C for molecular analysis. To determine the presence of morbillivirus, we obtained samples of cerebrum, cerebellum, lung, lymph node, and urinary bladder and applied immunohistochemistry techniques by using anti–canine distemper virus (monoclonal, 1:400; VMRD, Inc., https://vmrd.com) as described (*3*). We also examined organ samples by reverse transcription PCR, subjecting cerebrum, cerebellum, lungs, and lymph nodes to RNA extraction by using TRIzol LS Reagent (Thermo Fisher Scientific Inc., https://www.thermofisher.com) according to manufacturer instructions. We performed complementary DNA synthesis by using GoScript Reverse Transcriptase (Promega, https://www.promega.com) and semi-nested PCR for detecting the L gene of paramyxoviruses by using GoTaq DNA polymerase (Promega) (*4*). We performed conventional PCR to detect the P gene, according to a published protocol (*5*). We purified positive reactions with the PureLink PCR purification kit (Thermo Fisher Scientific) and determined sequences by using the Sanger method (ABI PRISM 3100 genetic analyzer, Big-Dye Terminator v.3.1 Cycle Sequencing Kit; Thermo Fisher Scientific). We assembled sequences by using Geneious Prime version 2022.2.1 (Dotmatics, https://www.dotmatics.com) and analyzed them through BLASTn (https://blast.ncbi.nlm.nih.gov). For phylogenetic analysis, we retrieved sequences from GenBank and aligned using ClustalW with MEGA version 11 software (https://www.megasoftware.net). Finally, we analyzed sequences through the maximum-likelihood method. 

MN1 was a juvenile male, 9.4 meters in length, with a poor body condition score. MN2 was a juvenile male, 11 meters in length, with a regular body condition score. Our microscopic investigations revealed findings primarily related to the central nervous system (CNS) (Figure, panels A, B; Appendix Table), as well as moderate lymphoid depletion in mesenteric and mediastinal lymph nodes in both specimens. Immunohistochemistry revealed discrete positive immunostaining in MN1 and marked positive immunostaining in MN2 for morbillivirus in astrocytes, neuronal cell bodies, and axons (Figure, panels C, D). In conducting reverse transcription PCR for MN1, we noted that the CNS tested positive for the L gene. Sequencing provided a 411-bp sequence (GenBank accession no. PP025976) that exhibited high similarity with the sequence of *Sotalia guianensis* morbillivirus (GD-CeMV; accession no. MG845553.1), showing 99.03% identity and 100% coverage (Appendix Figure 2, panel A). We saw different RT-PCR results in MN2, where all examined organs tested positive for the P gene but negative for the L gene. We obtained a 303-bp sequence of the P gene (GenBank accession no. PP549531), which exhibited 100% identity and 100% coverage with the reference GD-CeMV sequence (*6*) (Appendix Figure 2, panel B).

**Figure:**
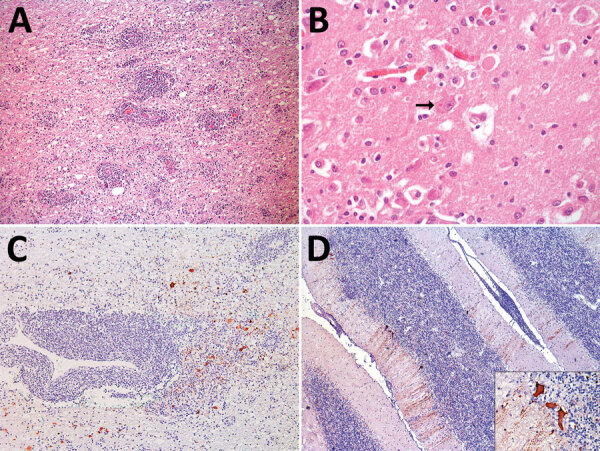
Microscopic findings of cetacean morbillivirus infection in 2 humpback whales in southern Brazil, 2022 (*Megaptera novaeangliae*). A) Cerebral cortex from whale MN2,. Note the pronounced perivascular cuffs composed of lymphocytes and plasma cells, moderate gliosis, and discreet vacuolization of the white matter. Hematoxylin and eosin stain; original magnification ×200. B) Cerebral cortex from whale. Eosinophilic intracytoplasmic inclusion body in a neuronal cell (arrow). Hematoxylin and eosin stain; original magnification ×400. C) Cerebral cortex from whale MN2. Neurons and astrocytes show severe, multifocal, cytoplasmic immunostaining with a marked perivascular lymphoplasmacytic cuff. Immunohistochemistry anti-canine distemper virus, morbillivirus; original magnification ×100. D) Cerebellum from MN2. Purkinje cells exhibit pronounced, multifocal, cytoplasmic immunostaining. Inset: Intense and granular immunostaining is observed in the cell body, in the dendrites of Purkinje cells, and occasionally in granule cells. Immunohistochemistry anti-canine distemper virus, morbillivirus; original magnification ×400.

There are reports of CeMV in various cetaceans, but few reports for mysticetes (*2*). Morbillivirus has been identified in fin whales (*Balaenoptera physalus*) and is associated with death and strandings. The main findings included CNS lesions and lymphoid depletion (*7*). In odontocetes, the main findings reported for GD-CeMV infection are CNS lesions and pneumonia (*6*). Our study observed alterations in the CNS (Appendix Table) and moderate lymphoid depletion in lymph nodes. The first record of GD-CeMV occurred in Brazil in 2014 in a Guiana dolphin (*Sotalia guianensis*) (*2*,*6*). A highly similar sequence was also found in respiratory samples from healthy humpback whale using real-time RT-PCR (*8*). A retrospective study identified GD-CeMV in southern right whales (*Eubalaena australis*) (*9*).

For MN1, we were able to amplify a fragment of the morbillivirus L gene through seminested PCR, which we confirmed by sequencing, but not for the P gene. For MN2, we could not amplify the L gene but did amplify the P gene using conventional PCR. Both L and P gene sequences are closely related to a similar sequence from a study conducted in 2018, in which not all tested cetaceans were positive for both genes (L and P) (*6*), supporting our findings. The humpback whale southwest Atlantic population follows its route between Antarctica and the Abrolhos Bank (northeast Brazil), which is distant from the southern coast of Brazil (*1*). Strandings in off-route areas and reduced body scores suggest a weak condition of the specimens (*1*,*2*,*6*,*7*), a theory supported by our findings.

Our findings for the 2 humpback whales we evaluated, combined with those from other CeMV-related studies, indicate that a highly related cluster of strains (GD-CeMV) is circulating in the southwestern Atlantic (*6*,*8*,*9*), as demonstrated by previous phylogeography (*10*). Considering the prior analysis of the partial P gene, CeMV strains are not restricted to specific regions because cetaceans are migratory and strains are not host specific (*10*). Our findings of nonsuppurative meningoencephalitis in these whales, caused by CeMV that shows similarity to GD-CeMV, provide evidence of this viral threat to these and other cetaceans.

Appendix Additional information for characterization of cetacean morbillivirus in humpback whales, Brazil.
