# Supplementary material for: Characterization of Cetacean Morbillivirus in Humpback Whales, Brazil
Source: Emerg Infect Dis. 2024 Jun;30(6):1296–8. doi: 10.3201/eid3006.231769 (PMC11138977; doi:10.3201/eid3006.231769)
Supplement: Appendix — Additional information for characterization of cetacean morbillivirus in humpback whales, Brazil. [file 23-1769-Techapp-s1.pdf]

Article DOI: http://doi.org/10.3201/eid3006.231769

EID cannot ensure accessibility for supplementary materials supplied by authors. Readers who have difficulty accessing supplementary content should contact the authors for assistance.

Characterization of Cetacean Morbillivirus in Humpback Whales, Brazil

Appendix

Appendix Table: Histopathologic alterations observed in the central nervous system of 2 stranded humpback whales (*Megaptera novaeangliae*) in southern Brazil in 2022.

	Lesion intensity	
Lesions	MN1	MN2
Gliosis	+++	+++
Mononuclear perivascular cuff (cerebrum)	+++	+++
Mononuclear perivascular cuff (cerebellum)	+	+++
Gitter cells	+++	++
Mononuclear meningitis (cerebrum)	+	+
Mononuclear meningitis (cerebellum)	+	++
Perivascular hemorrhage	+	+
Neuronal necrosis	+	+
Multifocal vacuolization of white matter	-	++
Intracytoplasmic/intranuclear inclusion bodies (cerebrum)	+	+

MN1: *Megaptera novaeangliae* specimen 1; MN2: *M. novaeangliae* specimen 2; Lesion intensity: + discrete; ++ moderate; +++ severe; - absent

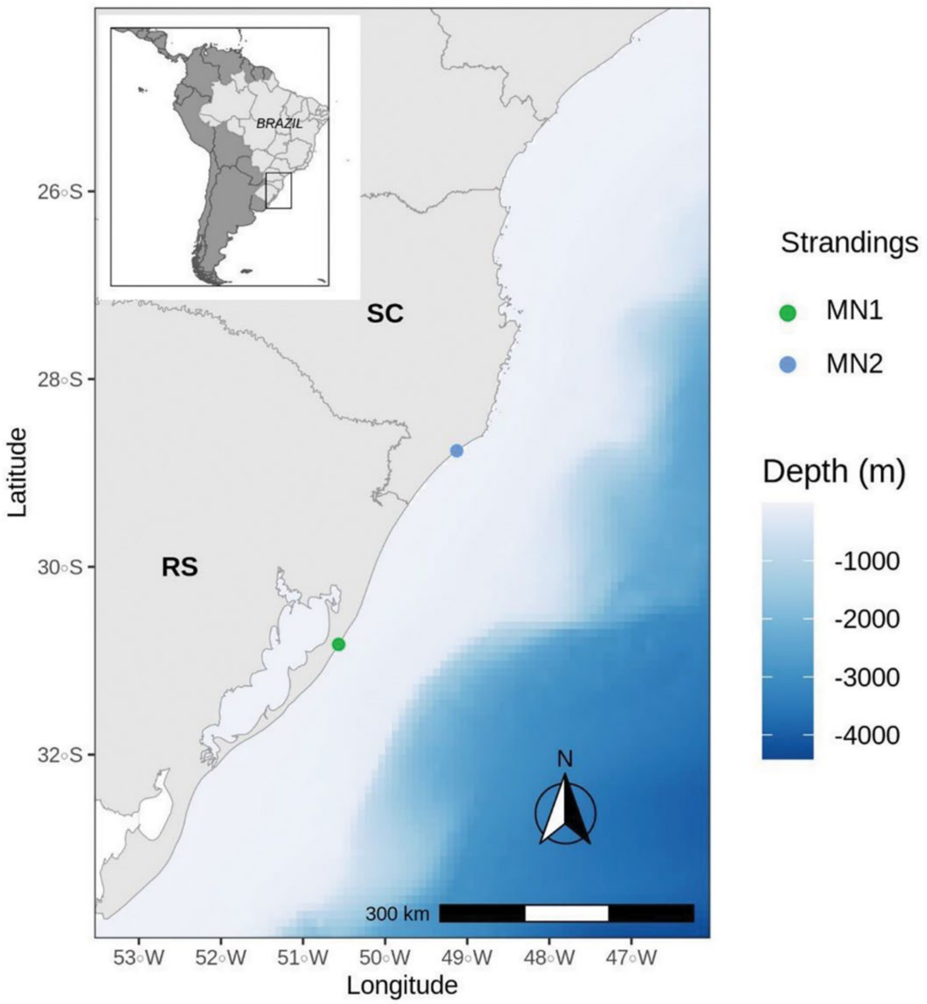

Appendix Figure 1. Map showing the location of 2 *Megaptera novaeangliae* strandings in southern Brazil in 2022. MN1: *M. novaeangliae* specimen 1; MN2: *M. novaeangliae* specimen 2; RS: Rio Grande do Sul State; SC: Santa Catarina state.

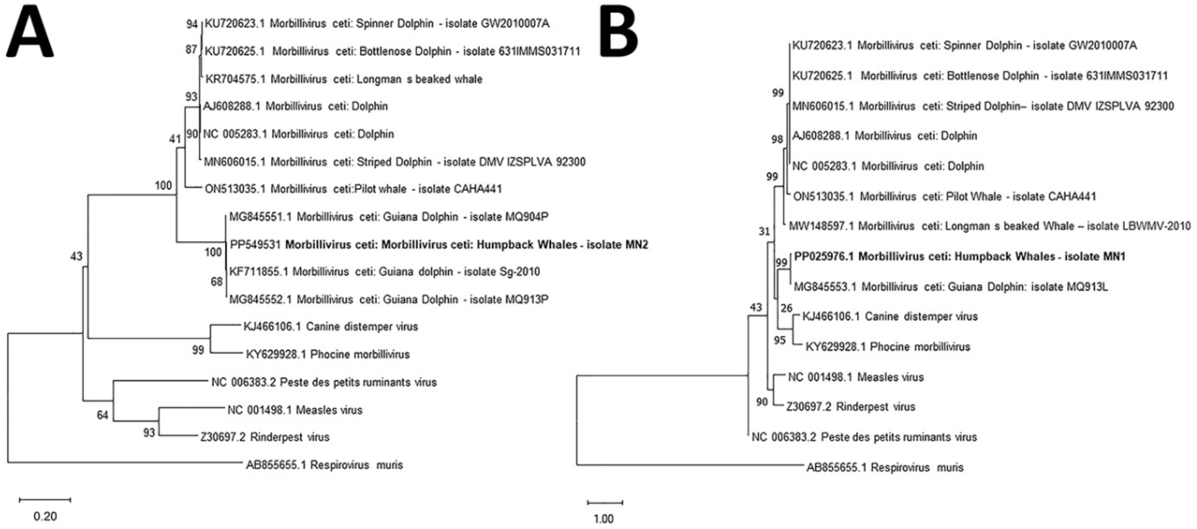

Appendix Figure 2. A) Phylogenetic analysis performed based on P gene sequence of representative species from morbillivirus genus. B) Phylogenetic analysis performed based on L gene sequence of representative species from morbillivirus genus. Analyses were conducted with 1000 bootstrap replicates. *Respirovirus muris* was added as an outside group. The sequence detected in the present study is highlighted.
